# Pediatric emergency room activities in Italy: a national survey

**DOI:** 10.1186/s13052-015-0184-9

**Published:** 2015-10-15

**Authors:** Riccardo Longhi, Raffaella Picchi, Domenico Minasi, Alessandra Di Cesare Merlone

**Affiliations:** Unità Operativa di Pediatria, Ospedale Sant’Anna, San Fermo della Battaglia, Como Italy; Unità Operativa di Pediatria, Ospedale di Polistena, Reggio Calabria Italy

**Keywords:** Short stay observation, S.S.O, Emergency units

## Abstract

**Background:**

In Italy, the number of accesses to the Emergency Units has been growing for the past 30 years. This, together with a low coordination between hospital and peripheral pediatric services, has brought to an unnecessarily high number of hospital admissions. For this reason, it is essential to plan and implement strategies able to improve the appropriateness of hospital admissions. In the ’90s, the Short Stay Observation was extended to pediatric patients. As highlighted by the report “Guidelines for Pediatric Observation Units” (2005), patients receive considerable benefits from a short hospital permanence. The purpose of the study is to report data about the Pediatric Emergency Room activities in Italy.

**Methods:**

In 2011, the Italian Society of Pediatrics promoted an online data collection to investigate organization and activity of Italian Pediatric and Neonatal Units. A form, containing 140 questions, was sent to 624 Pediatric and Neonatology Units. This study will be focused only on data regarding pediatric Emergency Rooms (E.R.) and Observation Units.

**Results:**

237 units replied, 183 if we focus on units with pediatric inpatient service. Based on the results, E.R Units were provided with a dedicated pediatrician in 56 % of the cases: of these, 85 % for 24 h. The majority of the patients were seen by a pediatrician. In only 8 % of the units, patients visited by a pediatrician were less than 40 %. The age limit was 14 years in 60 % of the cases. In 72 % of participating units a E.R. triage was carried out. Only 18 % of units registered more than 10000 E.R. visits/year. The percentage of children hospitalized after accessing the E.R. was significantly higher in southern regions (more than 20 % of the units hospitalized more than 40 % of children entering the E.R.). 66 % of the units were provided with an Observation Unit. In 61 % of the cases, the duration did not exceed 24 h. In more than half of the structures, less than 10 % of the E.R. visits went into observation. The type of remuneration was not homogeneous.

**Conclusions:**

The study highlights the heterogeneity of the Italian reality, with great possibilities for improvement, especially in southern regions.

## Background

In Italy, the number of Emergency Room (E.R.) admissions of adult and pediatric patients has been growing considerably over the past 30 years. In 2005, the Ministry of Health Advisory Committee launched the “Emergency System Improvement Plan”. At that time, nearly 50 million visits per year were registered. Pediatric patients represented the 10–15 % of the overall number [1].

Ninety percent of the cases were admissions, without there having been any previous contact with a medical practitioner. This being due to the fact that the highest concentration of admissions was registered during holidays and weekends. Moreover, data showed that most patients admitted to E.R. were coded either white or green (low degree of severity as opposed to yellow or red, high or very high severity).

Based on this information, it seems clear that the lack of interaction and cooperation between hospitals and family practitioners represents one of the main causes for the high E.R. admissions registered in the Country. There is, therefore, an urgent need to plan and implement new strategies i order to best utilize E.R. facilities.

In Italy, Short Stay Observation (S.S.O.) was extended to pediatric patients for the first time in 2003, for the purpose of continued care and treatment for patients in E.R., as well as reducing discomfort for both children and families, as highlighted by the report “Guidelines for Pediatric Observation Units” (2005) [2].

The aim of this study is to analyze the Italian Pediatric Emergency Room activities focusing our attention primarly on the S.S.O. service. This approach has the potential to become an extremely useful tool in decreasing and optimizing pediatric hospital admissions. To achieve this goal, we used the results of a national survey launched in 2011 and edited by the Italian Society of Pediatrics. The survey focused on the activity of Pediatric and Neonatal Departments in Italy, in 2010.

## Methods

In 2007, the Italian Society of Pediatrics promoted an online, multiple choice, data collection in order to investigate the organizational structure and activities of Pediatric and Neonatal Units. A specific form to collect informations was used.

Four years later, in 2011, the research process was repeated, this time focusing on the activity in 2010. Data collection took place from May until December 2011. The present study is based on the results of this last survey.

The form consisted of 140 questions concerning various hospital activities: from ordinary admissions to Day Hospital, Day-Surgery, E.R., S.S.O., Nursery, Neonatal Pathology, Neonatal Intensive Care Unit and Outpatient Clinics. This study will focus only on pediatric E.R. and S.S.O. activities.

Italy was divided into 3 macro areas: northern Italy (Emilia Romagna, Friuli Venezia Giulia, Liguria, Lombardy, Piemonte, Trentino Alto Adige, Valle d’Aosta and Veneto); central Italy (Abruzzo, Lazio, Marche, Tuscany and Umbria); southern Italy (Basilicata, Calabria, Campania, Molise, Puglia, Sardinia and Sicily).

The questionaire (Table 1) was sent to 624 Pediatric and Neonatology Units around the Country. 237 replied: 117 in northern Italy (49 %), 48 in central Italy (20 %), and 72 in southern Italy (31 %).

**Table 1:**
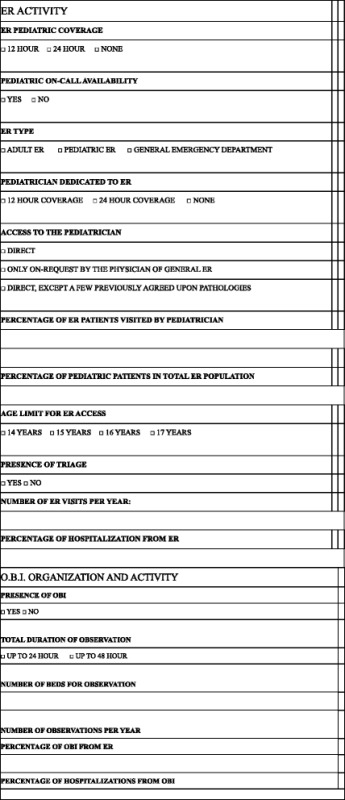
Extract of the questionnarie with the questions pertinent to this study

Statistical analysis was performed using the chi-square test considering significant a *p* value < 0,05.

Some data were processed utilizing linear regression test, in order to analyze a possible functional relationship between considered variables (in our study, the percentage of admissions from E.R. and the number of E.R. admissions).

## Results and discussion

### Response rate

Overall, when compared with the survey conducted in 2007 [3], there was a significant decrease in the percentage of responses, that dropped from 80 to 39 % (*p* < 0,001).

The survey showed the prevalence of Public Hospitals both in the group of the contacted facilities and in the group that actually participated in the survey: 84 and 83 %, respectively. University Hospitals represented respectively 8 and 11 %; Pediatric Hospitals 5 and 2 % and accredited Private Hospitals 2 and 4 %. Uncredited Private Hospitals represented 1 % of contacted units but their participation in the survey was null.

### Pediatrician coverage of ward and E.R

Almost 83 % of the units ensured a 24-h medical coverage (196), while 17 % (41) offered a 12-h coverage. In 75 % of the cases, (177) there was an operating on-call availability service. Table 2 illustrates the geographical distribution of these parameters with significant differences between northern and southern regions as reported (coverage h24 *p* 0.024; coverage h12 *p* 0.024; on-call availability service *p* 0.009).

**Table 2:** Distribution of the parameters for macro area, Italy and *p* value

	Parameters	North	Center	South	Italy	*P* value*
1	Coverage h24	90 % (105/117)	79 % (38/48)	77 % (56/72)	84 % (196/237)	0,0245c
2	Coverage h12	10 % (12/117)	21 % (10/48)	23 % (16/72)	16 % (41/237)	0,0245c
3	On call availability service	68 % (79/117)	77 % (37/48)	85 % (61/72)	75 % (177/237)	0,0088c
4	Pediatrician dedicated to E.R. h24	56 % (51/93)	47 % (17/36)	39 % (21/54)	48 % (89/183)	
5	Pediatrican dedicated to E.R. h12	12 % (11/93)	3 % (1/36)	4 % (2/54)	8 % (14/183)	
6	No pediatrician dedicated to E.R.	33 % (31/93)	50 % (18/36)	57 % (31/54)	44 % (80/183)	0,0044c
7	Direct access of all children to E.R. pediatrician	47 % (44/93)	39 % (14/36)	44 % (24/54)	45 % (82/183)	
8	Direct access to E.R. pediatrician except agreed diseases	30 % (28/93)	8 % (3/36)	6 % (3/54)	18 % (34/183)	0,010a
						0,00044c
9	Access to pediatrician only on request from E.R. physician	23 % (21/93)	53 % (19/36)	50 % (27/54)	37 % (67/183)	0,0010a
						0,0010c
10	E.R. triage	86 % (80/93)	83 % (30/36)	39 % (21/54)	72 % (131/183)	0,0010b
						0,0010c
11	Age limit for admission 14 years	52 % (48/93)	53 % (19/36)	80 % (43/54)	60 % (110/183)	0,007b
						0,0010c
11b	Age limit for admission 15 years	4 % (4/93)	0 % (0/36)	4 % (2/54)	3 % (6/183)	
12	Age limit for admission 16 years	9 % (8/93)	17 % (6/36)	9 % (5/54)	10 % (19/183)	
13	Age limit for admission 17 years	35 % (33/93)	30 % (11/36)	7 % (4/54)	26 % (48/183)	0,004b
						0,0010c
14	Presence of S.S.O.	80 % (74/93)	67 % (24/36)	43 % (23/54)	66 % (121/183)	0,025b
						0,0010c
15	S.S.O. fixed reimbursement	57 % (42/74)	67 % (16/24)	40 % (9/23)	55 % (67/121)	
16	S.S.O. reimbursement linked to health services provided	32 % (24/74)	21 % (5/24)	30 % (7/23)	32 % (36/121)	
17	S.S.O. reimbursement linked to disease type	11 % (8/74)	12 % (3/24)	30 % (7/23)	15 % (18/121)	0,02c
18	Length of S.S.O. less than 24 h	55 % (41/74)	58 % (14/24)	83 % (19/23)	61 % (74/121)	0,019c
19	Length of S.S.O. less than 48 h	45 % (33/74)	42 % (10/24)	17 % (4/23)	39 % (47/121)	0.019c

*P value for differences between north and center (a), center and south (b), north and south (c). Only significative P are recorded (*p* < 0,05)

**Fig. 1:**
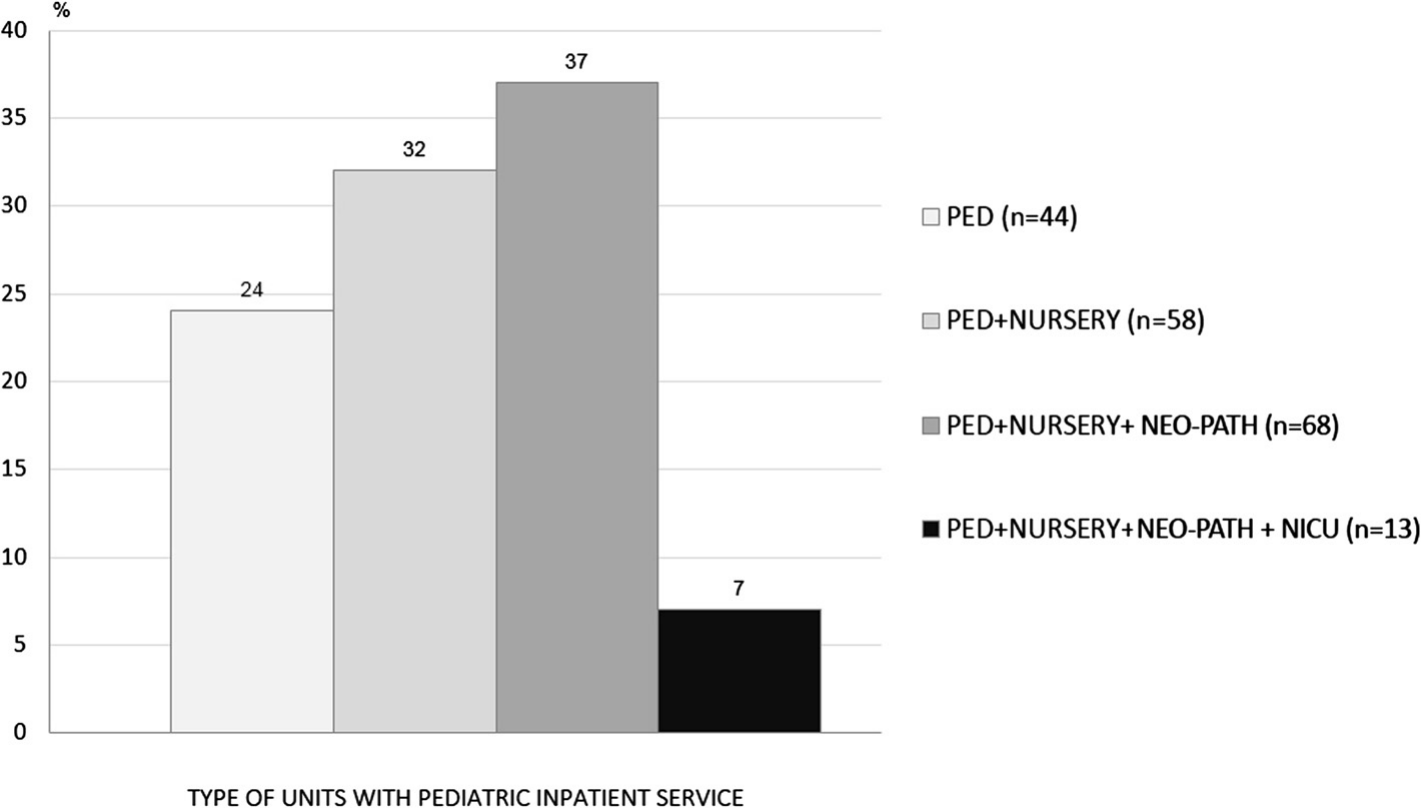
% distribution of facilities per type of units with pediatric inpatient service

**Fig. 2:**
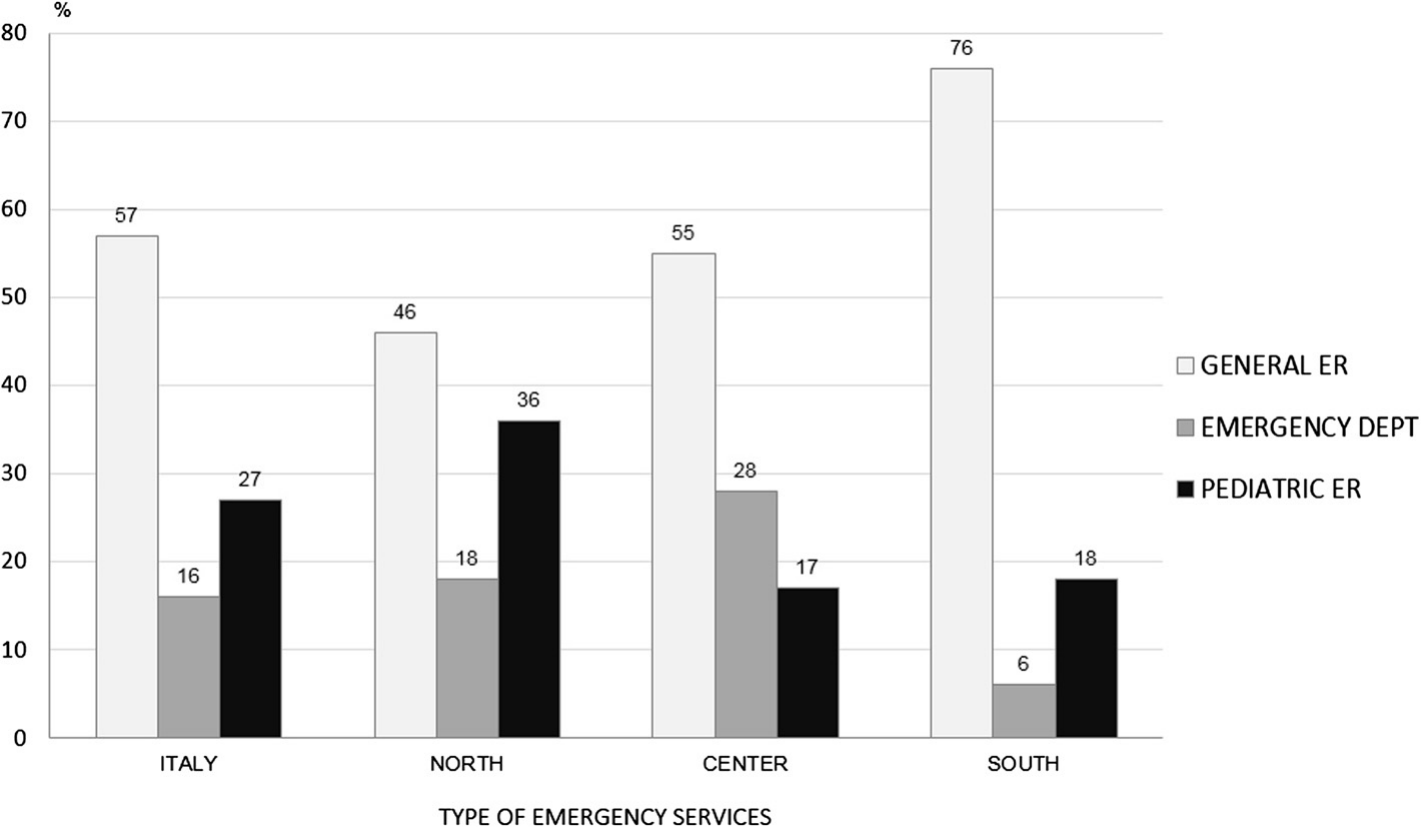
% distribution of facilities per type of emergency services: Italy and macro areas

Focusing on units with pediatric inpatient service (associated or not to neonatal activities), the number decreased from 237 to 183 (Fig. 1). 57 % (104) managed pediatric patients in adult E.R., 27 % (49) in pediatric E.R. and 16 % (30) in General Emergency Departments (DEA), with an uneven geographical distribution, as reported in Fig. 2.

There was a pediatrician assigned to the E.R. in 56 % of the units: in 85 % (89) of them for 24 h and in 15 % (14) only for 12 h during the day time.

In 45 % of the cases (82) access to the pediatrician was direct, in 37 % (67) only by the request of the general E.R. physician and in 18 % (34) it was direct, with the exception of a few previously agreed upon pathologies. Table 2 illustrates the geographical distribution of these parameters. In particular, it seems that in the E.R. of northern hospitals, the number of children seen directly by a pediatrician is significantly higher than in the rest of the Country (*p* < 0,001).

Altogether, the percentage of patients seen by a pediatrician varied. In more than 66 % of the cases the pediatrician saw approximately 80 % of the children entering the E.R. Conversely, in 8 % of the cases, the percentage of children seen by the pediatrician was less than 40 %. The age limit was 14 years in 60 % of the cases, 17 in 26 %, 16 in 11 % and 15 in 3 % of the cases, with significant differences between north and south regarding age limit for admission of 14 years old (*p* <0.001) and of 17 years old (*p* < 0.001) and between center and south (*p* 0.007 and *p* 0.004 respectively) as reported in Table 2.

### E.R. activity

**Fig. 3:**
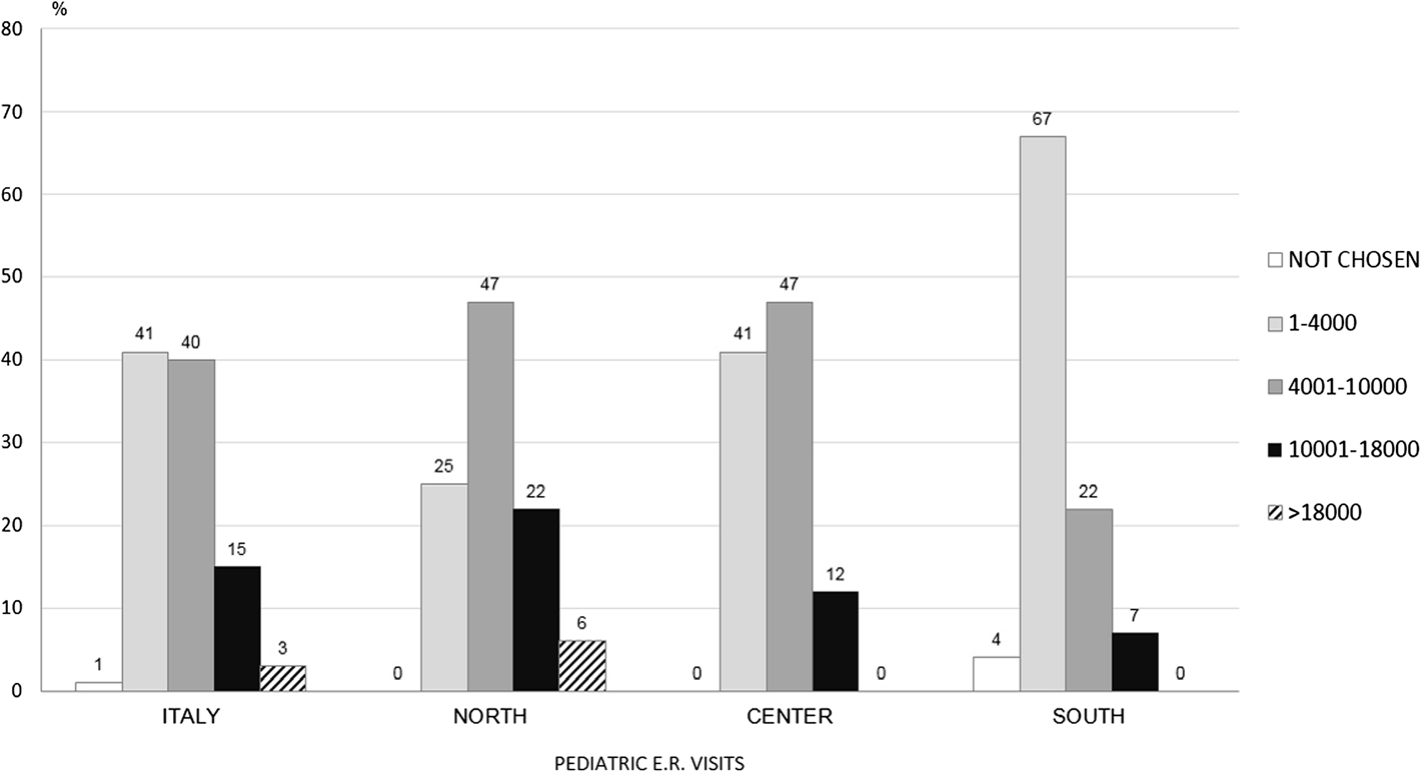
% distribution of facilities per pediatric E.R. visits: Italy and macro areas

In 72 % of the units (131), a triage was carried out, with a significant difference between northern and southern regions (*p* < 0.001) and between central and southern regions (*p* < 0.001) as reported in Table 2. Only 18 % of the facilities registered more than 10000 E.R. visits per year. The geographical distribution is shown in Fig. 3. Several units in the south registered a lower number of pediatric E.R. admissions (67 % of these facilities accepted less than 4000 pediatric patients per year). In terms of the actual percentage of children hospitalized after being admitted to the E.R., there was a huge difference between northern and southern Italy. In the north, 66 % of the units hospitalized up to 10 % of patients entering the E.R. vs 28 % and 20 % in the center and south, respectively (*p* < 0,001, north vs south; *p* 0,01, north vs center; p n.s. center vs south). Furthermore, in the south, more than 20 % of the facilities hospitalized more than 40 % of the children entering the E.R.

**Fig. 4:**
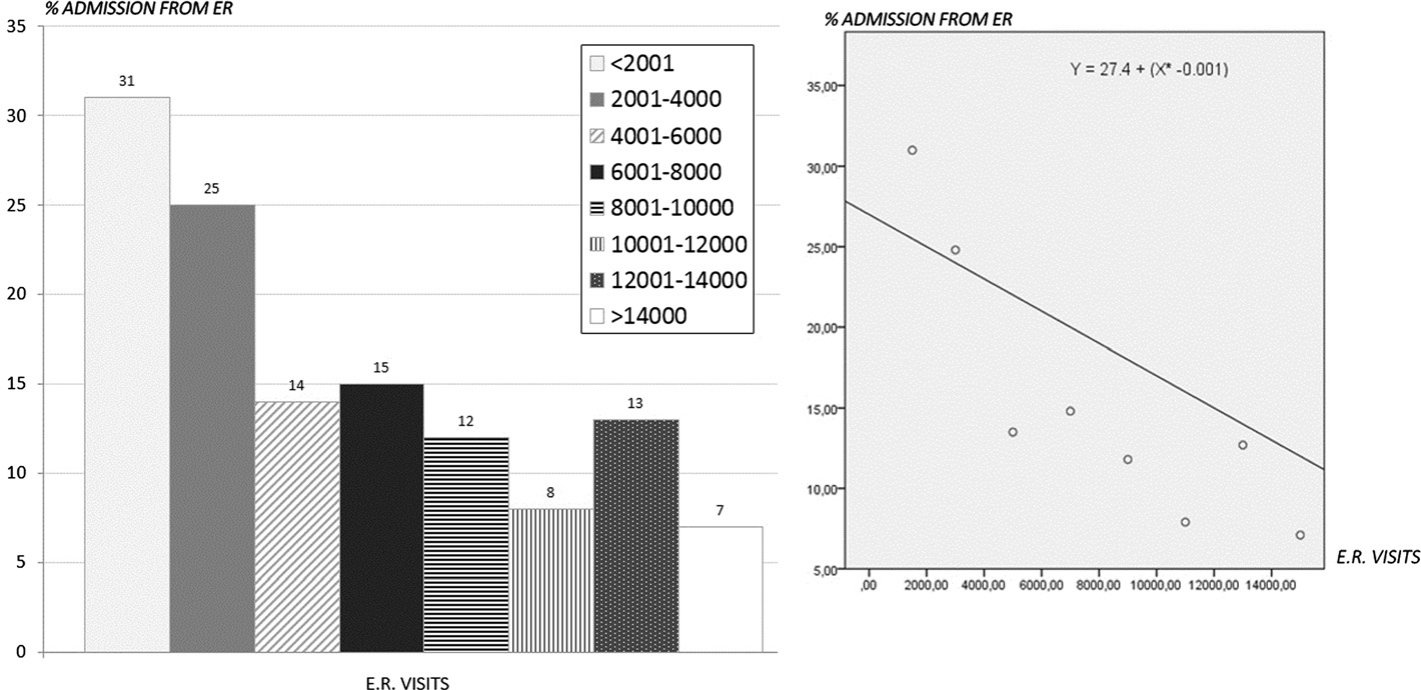
% distribution of admissions from E.R. per classes of numbers of E.R. visits (Italy) and regression line (*p* < 0,001)

Figure 4 combines the values of E.R. admissions with those of hospitalization rate through E.R. with significant difference (*p* < 0,001) between north and center and north and south and no significant difference between center and south.

### S.S.O. *organization and activity*

Sixty six percent (121) of the facilities were provided with an S.S.O. service. Again, the geographical distribution was not homogeneous: S.S.O. was available in 80 % of the northern units, 67 % of the central and only 43 % of the southern units with significant differences between northern and southern regions (*p* <0.001) and between center and southern regions (*p* 0.025) as reported in Table 2.

In 61 % of the facilities, the total duration of observation was limited to 24 h. In the remaining 39 %, the duration could last for up to 48 h. More specifically, in northern and central Italy, the number of observations with a length up to 24 h and those with a duration of up to 48 h were similar (55 % vs 58 % and 45 % vs 42 % respectively). In the south, most facilities (83 %) kept patients for up to 24 h (Table 2).

Seventy percent of the facilities were equipped with 1 or 2 beds, 24 % had 3 or 4 beds and 6 % were provided with more than 4 beds. The following statistics detail the volume of activity: approximately 12 % performed less than 100 observations per year; 21 % 100–250; 30 % 251–500; 21 % 501–1000; 16 % more than 1000.

## Discussion

The data supplied by this research contribute to the task of evaluating the current state of Emergency services in Italy and in planning future strategies for improvement.

Unfortunately, there was a significant drop off in response rates to the questionaire from about 80 % in 2007, to 39 % in 2011 (*p* < 0,001).

There are potentially many reasons behind this reduction. Among these, a great deal of recent senior personnel turnover in pediatric units resulted in more barriers in communicating and coordinating with the new Directors. Another factor was that a large amount of activity data, rather difficult to obtain, mandatory in 2011, were omitted in the previous research. Lastly, the increasing work overload may have made it harder to make time to complete the questionaire.

Despite the low response rate seeming to compromise the representativeness of the data obtained, the characteristics of responding hospital facilities accurately reflect reality, with the only notable exception being Pediatric Hospitals, which responded to a lesser degree.

Results show that 83 % of the units ensured an inpatient pediatric 24-h coverage, while 17 % a 12-h coverage with significant differences between northern and southern regions as represented in Table 2. In our opinion, these data are satisfactory, especially when compared with those of the previous survey (83 % vs 68 % and 17 % vs 18 % respectively) (p n.s.). More specifically, the 24-h pediatric coverage has increased from 75 to 90 % in the north (p n.s.); from 64 to 79 % in the centre (p n.s.); and from 59 to 77 % in the south (p n.s.). Furthermore, 56 % of the units were equipped with a pediatrician dedicated to the E.R. in 85 % of the cases for 24 h and in 15 % of cases for 12 h. In 48 % of units, pediatricians examined more than 90 % of children entering the E.R. Unfortunately, only in 27 % of the facilities was there a Pediatric E.R.. In our opinion, this represents a warning regarding the idea of the” specificity of the pediatric area”. This concept involves not only the proper and necessary separation of space from the adult population, but also the availability of a pediatric triage and nursing staff with specific expertise in pediatric care.

The data regarding the age limit pertaining to the pediatric area were absolutely unespected. In 2007, 40 % of the participating units claimed to have hospitalized patients under 17 years. In 2011, this percentage dropped to 26 % (p n.s.), while 60 % of the units limited the admission to 14 years old patients. All three macro areas have lowered the age of pediatric competence, but to a different extent: 80 % of southern facilities, for example, hospitalized patients only under 14 years, with a significant difference compared to northern facilities (*p* <0.001) and centre facilities (*p* 0.007) as shown in Table 2. The loss of competence toward the adolescents could be a serious concern for pediatricians and, more importantly, for that segment of the population.

Analysing the volume of E.R. activities, the data show that only 18 % of the units made more than 10000 admissions per year. Most importantly, a considerable number of southern hospitals carried out less than 4000 E.R. visits a year. This information cannot be overstated, as it represents one of the most important causes of the high rate of admissions from E.R. (Fig. 4). None of the northern units hospitalized more than 40 % of E.R. patients, while in the center and south, respectively 8 % and 21 % of the units hospitalized more than 40 % of E.R. patients (*p* < 0,0001, north vs south; *p* 0,02, north vs center and center vs south). Analogous results are obtained when we take into account the units that hospitalized between 25 % and 40 % of patients admitted to the E.R: 4 % in the north, 27 % in the centre and 36 % in the south (*p* < 0,0001, north vs south; *p* 0,0001, north vs centre; p n.s. centre vs south). There are no rational reasons to explain these differences. The only explanation stems from the need to achieve a quota of admissions to justify the existence of those units that, otherwise, should be reallocated. Based on these results, it is clear that high rates of hospitalization correspond to low numbers of E.R. admissions. Figure 4 shows that facilities with less than 2001 E.R. admissions per year hospitalized more than 30 % of patients vs 7,1 % of those making more than 14000 E.R. admissions per year. The regression analysis shows a negative correlation between the number of E.R. visits and the percentage of admission from E.R.: smaller and lesser specialized facilities seem to have a higher hospitalization rate (Fig. 4).

These results highlight an urgent need to rationalize the prospect of emergency services. In our opinion, facilities with low E.R. admissions should be converted into outpatient clinics. This would lead to a decrease in the number of patients hospitalized, without compromising the quality and safety of care. In fact, the great majority of patients with serious medical problems would inevitably be directed to units providing 24-h pediatric coverage and equipped with an adequate number of pediatricians [4–7].

As pertaining to S.S.O., this assistance model was adopted by 66 % of the participating units. Once again, the geographical distribution was not homogeneous. Almost 43 % of the southern units offered S.S.O. services vs 67 % of the central and 80 % of the northern ones. Statistical analysis confirmed significant differences between northern and southern regions (*p* <0.001) and between central and southern regions (*p* 0.025) (Table 2).

As for the S.S.O. time limit, it was less than 24 h in 61 % of centers (55 % in northern regions, 58 % in central and 83 % in those southern), less than 48 h in 39 % of centers (45 % in the north, 42 % in the centre and 17 % in the south). These data seem appropriate. In fact, in the United States, about one-third of pediatric patients hospitalized had a length of stay up to 48 h, and therefore, it would have been manageable in S.S.O. [8–10]. These data become even more significant when, in addition to focusing on the length of stay, patients are classified by their diseases. For some diseases (asthma, croup), the percentage of hospitalizations “avoided” and “saved” reached 70 % [11–13]. Analyzing the ratio between the number of short stay observations and E.R. admittances, we realize that in more than half of the cases, this ratio was less than 10 %, a number probably well below a reasonable level [8–11]. On the other hand, if we consider hospital admission rates after observation, data are in line with expectations: less than 20 %, in 61 % of cases. Unfortunately, in 25 % of the units, the admission rate was up to 21–40 %, and in 10 % it even exceeded 40 %. As reported in one of the most extensive studies [10], the expected admission rate is about 20 %. We believe that percentages higher than the expected level indicate a need for a stricter adherence to the S.S.O. admission criteria. In other words, this healthcare model should be more rigorously regulated through the implementation of protocols planned to better define and clarify appropriate use and correct diagnostic and therapeutic criteria for each disease. We think that, when properly used, the S.S.O. model is an efficient tool to reduce inappropriate E.R. discharges and to increase patient safety and comfort. Furthermore, if correctly formalized, S.S.O. could lead to a rationalization of the National Health System’s resources [14–16]. In fact, it could potentially reduce inappropriate hospital admissions, considering that many pediatric admissions last for less than 48 h. These admissions increase the saturation of pediatric wards, take away resources more appropriately used elsewhere and sometimes force patients to stay in unsuitable environments or require a demanding and risky transfer to another hospital.

## Conclusions

Our data highlight the heterogeneity of the Italian situation. Even though the moral wealth of our Country comes from a balanced combination of different cultures and traditions, when it comes to healthcare, we believe in the need for a wider homogeneity and uniformity. This could be accomplished by better regulating pediatric activities inside and outside of the hospital. This is not meant to trivialize physicians’ functions and responsibilities with the strict adoption of protocols and procedures, but signifies the necessity to provide the medical staff with the appropriate tools to perform his work in a more correct and safer way.
